# Prevalence and factors for anxiety and depression among secondary school teachers from Sfax city of Tunisia in times of the coronavirus disease 2019 pandemic: a cross-sectional study

**DOI:** 10.11604/pamj.2024.47.27.39550

**Published:** 2024-01-23

**Authors:** Nour Regaieg, Lobna Zouari, Yosra Mejdoub, Oumayma Elleuch, Najeh Smaoui, Rim Feki, Imen Gassara, Manel Maalej Bouali, Nada Charfi, Jihen Ben Thabet, Mohamed Maalej, Sana Omri

**Affiliations:** 1 Psychiatry “C” Department, Hedi Chaker University Hospital, Sfax, Tunisia,; 2 Epidemiology Department, Hedi Chaker University Hospital, Sfax, Tunisia

**Keywords:** Teachers, COVID-19, anxiety, depression

## Abstract

**Introduction:**

teachers have to adapt during the coronavirus disease 2019 (COVID-19) pandemic to many changes that could potentially make them more vulnerable to psychological distress. We aimed to assess anxiety and depression in Tunisian secondary school teachers during the COVID-19 pandemic and to explore their associated factors.

**Methods:**

we conducted a cross-sectional survey concerning Tunisian secondary school teachers between May 1^st^, 2021, and June 30^th^, 2021, using an online survey platform via Google Forms. Participants were asked to fill in a form including two psychometric tests: the General Anxiety Disorder 7 (GAD-7) and the Patient Health Questionnaire 9 (PHQ-9). We performed both univariable and multivariable logistic regression analyses.

**Results:**

a total of 170 secondary school teachers were included, of whom 22.4% (n=38) were males and 77.6% (n=132) were females. The median age was 45.5 years (Q1=39, Q3=49). The overall anxiety prevalence was 34.7% (n=59) while it was 41.7% (n=71) for depression. In multivariable analysis, anxiety was strongly associated with sleep disturbances (aOR: 5.1; 95% CI: 1.80-14.45; p=0.002) and depression (aOR: 33.91; 95% CI: 12.32-93.33; p<0.001) while depression was strongly associated with dissatisfaction with working conditions (aOR: 3.99; 95% CI: 1.49-10.65; p=0.006), the irregular wearing of protective masks (aOR: 3.94; 95% CI: 1.33-11.66; p=0.013) and anxiety (aOR=51.63; 95% CI: 17.74-150.25; p<0.001).

**Conclusion:**

secondary school teachers in Tunisia are characterized by a high rate of anxiety and depressive disorders which are related to personal and professional factors on which we can act by supplying of educational institutions by protective masks, the implementation of programs for adjusting working conditions and the practice of non-pharmacological interventions for insomnia management.

## Introduction

Human-to-human transmission of the 2019 coronavirus disease (COVID-19) has posed a major global health threat [1]. Finally, the World Health Organization (WHO) declared the outbreak of the COVID-19 infection as a Public Health Emergency of International Concern (PHEIC) on January 30^th^ [2] and a pandemic in March 2020 [3]. In Tunisia, from 3 January 2020 to 7 October 2022, there have been 1,145,829 confirmed cases of COVID-19 with 29,254 deaths, reported to WHO [4].

The increasing number of suspected and confirmed cases caused by COVID-19 created a climate of alarm and fear due to the very real threat of contraction and potential manifestation of the disease [5]. Therefore, apart from the danger of serious disease and possible mortality due to the COVID-19 infection, the new pandemic has also brought a large proportion of the global population face to face with an unprecedented emotional strain, resulting in panic attacks, stress disorders and feelings of depression [6,7] mainly due to the uncertainty they bring and the sudden changes in daily life they result in [5,6].

Several international studies researching the emotional impact of the COVID-19 pandemic on health professionals and vulnerable population groups in general, have been conducted [8,9]. However, there have been few researches on the emotional impact on teachers, especially in the Tunisian literature. In fact, COVID-19 is the greatest challenge that the expanded national education systems have ever faced. Teachers, apart from having a challenging and stressful profession [1], have to adapt during this pandemic to many other changes that could potentially make them more vulnerable to psychological distress such as anxiety and depressive disorders. More importantly, anxiety may increase the risk of cardiovascular disease, cancer, and even mortality [10]. Moreover, studies have shown that teachers´ stress and negative emotions can result in poor classroom performance [11] and impair their capacity to help and respond to students correctly [8]. Hence, a timely understanding of teachers´ emotional state is an urgent necessity. Therefore, we aimed in the current study to assess the psychological distress of Tunisian secondary school teachers during the COVID-19 pandemic, in terms of anxiety and depression and to explore their factors.

## Methods

**Study design:** we conducted a descriptive and analytical cross-sectional study to assess anxiety and depression in Tunisian secondary school teachers during the epidemic of COVID-19.

**Study setting:** the study was conducted in the period of time between May 1^st^, 2021, and June 30^th^, 2021. Data collection was carried out using an anonymous self-administered questionnaire through an online survey platform via Google Forms. The online survey began with details of the study´s purpose and requirements followed by an informed consent form with an explanation of the anonymity of participants and the confidentiality of all data obtained. Only after providing informed consent were participants able to continue the online survey. The reason for adopting an online survey was that it was difficult for researchers to collect data in person due to the epidemic situation.

**Participants:** of the list of 18 high schools and 33 middle schools of the public sector in Sfax city of Tunisia, 22 institutions had Facebook groups. The questionnaire was distributed to these groups of teachers. As the survey was posted online, only those who completed the questionnaire and clicked the submission were counted.

**Variables:** the form was designed to collect the personal and professional characteristics of the participants and included psychometric assessments. The characteristics of the teachers consisted of information about age, gender, education level, and marital status. Additional questions concerning the profession (professional seniority, workload, satisfaction with the working conditions, satisfaction with the online teaching) were requested. Finally, participants were asked about the history of COVID-19 attack and the repercussions of COVID-19 infection (sleep disturbances or suicidal thoughts since the advent of COVID-19 pandemic) as well as the adoption of COVID-19 preventive measures (regular mask wearing at work, registration on the vaccination platform, reception of the COVID-19 vaccination and otherwise, cause of the non-vaccination, belief that wearing a mask blocks the transmission of information or emotions between teacher and student).

**Data sources and measurement:** to assess anxiety and depression, we used two psychometric tests: the General Anxiety Disorder 7 (GAD-7) and the Patient Health Questionnaire 9 (PHQ-9).

**General Anxiety Disorder 7 (GAD-7):** the GAD-7 is a brief self-report scale that aims to assess generalised anxiety. Each of its 7 items is given a score ranging from 0 (not at all) to 3 (nearly every day). The total GAD-7 scale score ranges from 0 to 21. Cut points of 5, 10, and 15 might be interpreted as representing mild, moderate, and severe levels of anxiety. A GAD-7 score ≥ 10 has a sensitivity of 89% and a specificity of 82% to indicate generalised anxiety disorder when formally tested against the diagnostic psychiatric interviews [12].

**Patient health questionnaire 9 (PHQ-9):** the PHQ-9 is a shorter nine-item version of the complete PHQ. This self-report scale was developed to assess depression symptoms. The PHQ-9 is made up of the nine criteria used in the Diagnostic and Statistical Manual of Mental Disorders- Fourth Edition (DSM-IV) to diagnose depressive disorders. Each of the nine items can be scored from 0 (not at all) to 3 (nearly every day). The total scale score ranging from 0 to 27 reflects the severity of symptoms: PHQ-9 scores of 5-9 indicate mild, 10-14: moderate, 15-19: moderately severe, and ≥ 20: severe depressive symptoms. This questionnaire has been formally validated against structured diagnostic interviews performed by a mental health professional; PHQ-9 score ≥10 has a sensitivity of 88% and a specificity of 88% in the detection of major depression [13].

**Bias:** our study would include a sampling bias as not all the high and middle schools of the public sector of Sfax city had Facebook groups. Therefore, the representativeness of our sample could have been reduced.

**Study size:** a total of 170 secondary school teachers who answered the online questionnaire during the study period were included in the study.

**Quantitative variables:** in order to compare subjects, we divided them into two groups: with and without anxiety, and with and without depression.

**Statistical methods:** the analyses were performed using the Statistical Package for Social Sciences IBM version 23.0 (IBM SPSS Statistics, New York, United States). We performed both univariable and multivariable logistic regression analyses. Descriptive statistics were performed to explore the characteristics of the population. The severity of symptoms of anxiety or depression was determined according to the scores of different scales. Categorical data were presented as frequency (%). For the quantitative variables, we checked the normality of the distribution using the Kolmogorov-Smirnov test and the Shapiro-Wilk test. An estimate of the means with their standard deviations and of the median with quartiles (Q1, Q3) was thus carried out.

The comparison of the means on unpaired series was made by the test of “Student” when the distribution was normal and by the test of “Mann-Whitney” when the distribution was not normal. The comparison of two frequencies was done by the “Chi-square” test when the application conditions were verified, and by Fisher's test otherwise. Depending on the distribution of the parameters, correlations were performed either by the Pearson's r correlation test or by the Spearman's test. Multivariable logistic regression models were used to estimate odds ratios (OR) with 95% confidence intervals (CI) for potential factors of anxiety or depression. We retained a risk of error of 20% to include the indicator variables in the multivariate analysis. The significance level was set at 5% for both univariable and multivariable analyses.

## Results

**General characteristics of the study population:** a total of 170 secondary school teachers were included in this study. Thirty-eight participants (22.4%) were males and 132 (77.6%) were females. The median age was 45.5 years (Q1=39, Q3=49). The majority of teachers (82.9%, n=141) have registered on the vaccination platform, while 77.1% (n=131) have already received a COVID-19 vaccination. The causes of non-reception of the vaccination in 22.9% of teachers (n=39) were: not receiving a vaccination appointment (50%, n=18), fear of adverse effects (25%, n=9), an attack by the COVID-19 having coincided with the vaccination appointment (16.7%, n=6), doubt about the ineffectiveness of the vaccine (11.9%, n=4), absence of disease risk factors (13.9%, n=5) and pregnancy (3.3%, n=1). Table 1 shows the characteristics of participants according to the presence or absence of anxiety (or depression).

**Table 1:** general characteristics of the study population by anxiety and depression status

Characteristics	All participants (n=170)	Without anxiety (n =111)	With anxiety (n =59)	P-value	Without depression (n=99)	With depression (n=71)	P-value
**Gender (%)**				**0.005**			**0.01**
Male	38 (22.4)	32 (84.2)	6 (15.8)		29 (76.3)	9 (23.7)	
Female	132 (77.6)	79 (59.8)	53 (40.2)		70 (53)	62 (47)	
**Age**							
Age in years	45.5: Q1=39 Q3=49	44.15: Q1=8 Q3=28	41.8: Q1=8 Q3=38	0.08^2^	44.26: Q1=8 Q3=38	42.04: Q1=8 Q3=23	0.08^2^
< 45 years	98 (57.6)	59 (60.2)	39 (39.8)	0.1	53 (54.1)	45 (45.9)	0.2
≥ 45 years	72 (42.4)	52 (72.2)	20 (27.8)		46 (63.9)	26 (36.1)	
**Education status (%)**				0.39			0.38
Bachelor's degree	111 (65.3)	75 (67.6)	36 (32.4)		62 (55.9)	49 (44.1)	
Higher diploma	59 (34.7)	36 (61)	23 (39)		37 (62.7)	22 (37.3)	
**Married status (%)**				0.59			0.23
Married	114 (67.1)	76 (66.7)	38 (33.3)		70 (61.4)	44 (38.6)	
Non married	56 (32.9)	35 (62.5)	21 (27.5)		29 (51.8)	27 (48.2)	
**Professional seniority**							
Professional seniority in years	16:Q1=10.75 Q3=23	16: Q1=10 Q3=24	16: Q1=11 Q3=20	0.47^2^	17: Q1=10 Q3=24	16: Q1=11 Q3=20	0.37^2^
≤20 years	120 (70.6)	75 (67.6)	45 (76.3)	0.23	65 (65.7)	55 (77.5)	0.09
>20 years	50 (29.4)	36 (32.4)	14 (23.7)		34 (34.3)	16 (22.5)	
**Workload (%)**				**0.005^1^**			**0.018**
Low to medium	97 (57.1)	72 (74.2)	25 (25.8)		64 (66)	33 (34)	
High	73 (42.9)	39 (53.4)	34 (46.6)		35 (47.9)	38 (52.1)	
**Satisfaction with working conditions (%)**				**0.001^1^**			**< 0.001**
Dissatisfied	52 (30.6)	24 (46.2)	28 (53.8)		17 (32.7)	35 (67.3)	
Satisfied or very satisfied	118 (69.4)	87 (73.7)	31 (26.3)		82 (69.5)	36 (30.5)	
**Satisfaction with online teaching (%)**				0.71			0.74
Low to medium	162 (95.3)	105 (64.8)	57 (35.2)		93 (57.4)	69 (42.6)	
High	8 (4.7)	6 (75)	2 (25)		6 (75)	2 (25)	
**History of COVID-19 attack (%)**				0.1			0.53
Yes	53 (31.2)	30 (56.6)	23 (43.4)		29 (54.7)	24 (45.3)	
No	117 (68.8)	81 (69.2)	36 (30.8)		70 (59.8)	47 (40.2)	
**Sleep disturbances (%)**				**<0.001**			**< 0.001**
Yes	96 (56.5)	45 (46.9)	51 (53.1)		39 (40.6)	57 (59.4)	
No	74 (43.5)	66 (89.2)	8 (10.8)		60 (81.1)	14 (18.9)	
**Suicidal thoughts (%)**				**0.001**			**0.003**
Yes	19 (11.2)	6 (31.6)	13 (68.4)		5 (26.3)	14 (73.7)	
No	151 (88.8)	105 (69.5)	46 (30.5)		94 (62.3)	57 (37.7)	
**Regular mask-wearing at work (%)**				0.82			**0.04**
Yes	137 (80.6)	90 (65.7)	47 (34.3)		85 (62)	52 (38)	
No	33 (19.4)	21 (63.6)	12 (36.4)		14 (42.4)	19 (57.6)	
**Thought that wearing a mask blocks the transmission of information and emotions between teacher and student (%)**				**0.009**			**0.035**
Yes	141 (82.9)	86 (61)	55 (39)		77 (54.6)	64 (45.4)	
No	29 (17.1)	25 (86.2)	4 (13.8)		22 (75.9)	7 (24.1)	
**Registration on the vaccination platform (%)**				0.83			0.38
Yes	141 (82.9)	91 (64.5)	50 (35.5)		80 (56.7)	61 (43.3)	
No	29 (17.1)	20 (69)	9 (31)		19 (65.5)	10 (34.5)	
**Reception of the COVID-19 vaccination (%)**				0.85			0.63
Yes	131 (77.1)	86 (65.6)	45 (34.4)		75 (57.3)	56 (42.7)	
No	39 (22.9)	25 (64.1)	14 (35.9)		24 (61.5)	15 (38.5)	

Data are presented as median (25th - 75th percentile) or number (percentage); ^1^: P-value is from Fisher’s test for categorical variables; ^2^: P-value is from Mann-Whitney U test for continuous variables; The bold text is used to indicate the statistically significant p-values at alpha 0.05

**Figure 1:**
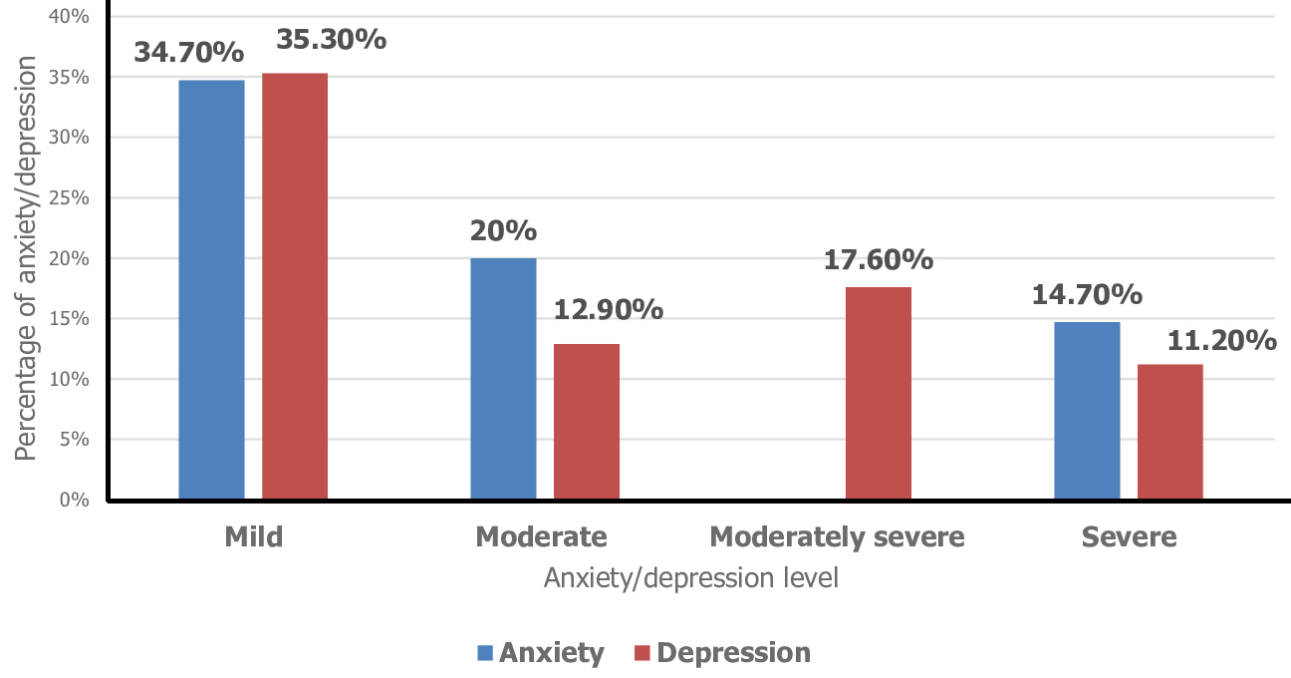
anxiety and depression levels among secondary school teachers during the COVID-19 epidemic in Tunisia

**Prevalence and different levels of anxiety and depression in the participants:** during the COVID-19 pandemic, the overall anxiety prevalence in our sample of Tunisian secondary school teachers was 34.7% while the overall depression prevalence was 41.7% according to the GAD-7 and PHQ-9. The severity levels of anxiety and depression in participants are shown in Figure 1.

## Analytical study

**Positive or risk factors for anxiety and depression according to univariable analysis:** as compared to participants without anxiety, participants with anxiety were different from the proportion of gender (p=0.005), workload (p=0.005), satisfaction with working conditions (p=0.001), sleep disturbances (p<0.001), suicidal thoughts (p=0.001), and thought that wearing a mask blocks the transmission of information and emotions between teacher and student (p=0.009). Nevertheless, our findings showed no association of anxiety with age (p=0.08), educational level (0.39), professional seniority (0.47), and vaccination status (0.85). Furthermore, we found that depression was significantly associated with female gender (p=0.01), a high workload (p=0.018), dissatisfaction with working conditions (p<0.001), sleep disturbances (p<0.001), suicidal thoughts (p=0.003), irregular wearing of protective masks (p=0.04) and thought that wearing masks blocks the transmission of information and emotions between teacher and student (p=0.035). The presence of depression was also significantly associated with the presence of anxiety (p<0.001). According to the Spearman test, the two scores GAD-7 and PHQ-9 were strongly correlated with each other (p<0.001; r=0.84). However, no association of depression was observed with age (p=0.08), educational level (0.8), professional seniority (0.37), and vaccination status (0.63).

Table 1 summarizes the correlation analysis between the characteristics of the participants and scores of GAD-7 and PHQ-9.

**Positive or risk factors for anxiety and depression according to multivariable analysis:** after adjusting for age, gender, workload, satisfaction with the working conditions, history of COVID-19 attack, insomnia, suicidal ideation, depression, and thought that wearing masks blocks the transmission of information and emotions, the highly predictive factors of anxiety were the presence of sleep disturbances (aOR: 5.1; 95% CI: 1.80-14.45; p=0.002) and depression (aOR: 33.91; 95% CI: 12.32-93.33; p<0.001). The results are illustrated in Table 2.

**Table 2:** odds of anxiety-related factors among secondary school teachers during the COVID-19 epidemic in Tunisia

Variable	Anxiety			
	Unadjusted OR (95% CI)	P-value	Adjusted OR (95% CI)	P-value
**Gender**				
Female	3.57 (1.39-9.14)	0.008	1.34 (0.26-6.93)	0.72
**Age**				
≤45 years	0.96 (0.93-1.00)	0.08	1.78 (0.47-6.73)	0.39
**Workload**				
High	2.51 (1.31-4.79)	0.005	2.13 (0.70-6.43)	0.17
**Satisfaction with working conditions**				
Dissatisfied	0.30 (1.65-6.47)	0.001	0.78 (0.25-2.36)	0.66
**Sleep disturbances**				
Yes	9.35 (4.05-21.57)	<0.001	5.1 (1.80-14.45)	**0.002**
**Suicidal thoughts**				
Yes	4.94 (1.77-13.81)	0.002	3.08 (0.71-13.25)	0.13
**Thought that wearing a mask blocks the transmission of information and emotions between teacher and student**				
Yes	0.25 (0.08-0.75)	0.01	0.079 (0.83-26.52)	0.07

CI: confidence interval; OR: odds ratio; adjusted OR: for age, gender, workload, satisfaction with working conditions, thought that wearing a mask blocks the transmission of information and emotions between teacher and student, insomnia, suicidal ideation and depression; the bold text is used to indicate the statistically significant p-values at alpha 0.05

After adjusting for age, gender, workload, satisfaction with working conditions, mask-wearing, thought that wearing masks blocks the transmission of information and emotions, insomnia, suicidal ideation, and anxiety, the highly predictive factors of depression were dissatisfaction with working conditions (aOR: 3.99; 95% CI: 1.49-10.65; p=0.006), irregular wearing of protective masks (aOR: 3.94; 95% CI: 1.33-11.66; p=0.013) and the presence of anxiety (aOR=51.63; 95% CI: 17.74-150.25; p<0.001). The results are shown in Table 3.

**Table 3:** anxiety and depression levels among secondary school teachers during the COVID-19 epidemic in Tunisia

Variable	Depression			
	Unadjusted OR (95% CI)	P-value	Adjusted OR (95% CI)	P-value
**Gender**				
Female	2.85 (1.25-6.49)	0.012	1.41 (0.37-5.32)	0.60
**Age**				
≤45 years	0.66 (0.35-1.24)	0.20	0.36 (0.08-1.52)	0.16
**Workload**				
High	2.10 (1.13-3.92)	0.019	1.43 (0.49-4.11)	0.50
**Satisfaction with working conditions**				
Dissatisfied	4.69 (2.33-9.43)	<0.001	3.99 (1.49-10.65)	0.006
**Sleep disturbances**				**0.08**
Yes	6.26 (3.07-12.74)	<0.001	2.49 (0.89-6.96)	
**Suicidal thoughts**				
Yes	4.61 (1.57-13.50)	0.005	1.15 (0.24-5.43)	0.85
Regular mask-wearing at work				
Yes	2.21 (1.02-4.80)	0.04	3.94 (1.33-11.66)	0.013
**Thought that wearing a mask blocks the transmission of information and emotions between teacher and student**				
Yes	2.61 (1.04-6.50)	0.03	0.42 (0.09-1.87)	0.25
**Anxiety**				
Yes	45.63 (17.06-122.04)	<0.001	51.63 (17.74-150.25)	<0.001

CI: confidence interval; OR: odds ratio; adjusted OR: for age, gender, workload, satisfaction with working conditions, regular mask wearing at work, thought that wearing a mask blocks the transmission of information and emotions between teacher and student, insomnia, suicidal ideation, and anxiety; the bold text is used to indicate the statistically significant p-values at alpha 0.05

## Discussion

We were interested through this study to assess the psychological distress of Tunisian secondary school teachers during the COVID-19 pandemic, in terms of anxiety and depression and to explore their factors. We found that 34.7% of teachers had moderate to severe anxiety while 41.7% had depression, with 11.2% exhibiting severe symptoms. Both anxiety and depression were statistically associated with the female gender (p=0.005 and p=0.01, respectively), a high workload (p=0.005 and p=0.018, respectively), sleep disturbances (p<0.001 and p<0.001, respectively), suicidal thoughts (p=0.001 and p=0.003, respectively), dissatisfaction with working conditions (p=0.001 and p<0.001, respectively) as well as with thought that wearing a mask blocks the transmission of information and emotions between teacher and student (p=0.009 and p=0.035, respectively). Furthermore, depression was significantly associated with irregular wearing of protective masks (p=0.04). The presence of depression was also significantly associated with the presence of anxiety (p<0.001). In multivariable analysis, anxiety was associated with sleep disturbances (aOR: 5.1; 95% CI: 1.80-14.45; p=0.002) and depression (aOR: 33.91; 95% CI: 12.32-93.33; p<0.001) while depression was associated with dissatisfaction with working conditions (aOR: 3.99; 95% CI: 1.49-10.65; p=0.006), the irregular wearing of protective masks (aOR: 3.94; 95% CI: 1.33-11.66; p=0.013) and anxiety (aOR=51.63; 95% CI: 17.74-150.25; p<0.001).

The rates of anxiety and depression observed in our study approach those reported by Stachteas *et al*. [14] who demonstrated that 34% of secondary school teachers in Greece felt anxious and very anxious during the pandemic, while 8% showed severe depressive emotions. Along with our findings, the study of Sigursteinsdottir *et al*. [15] revealed that primary school teachers in 2021 reported poor mental health with an increase of the incidence of sadness from 32% in 2019 to 47.9% in 2021. Additionally, according to a recent systematic review of studies carried out in China, Brazil, the United States of America, India, and Spain [16], the prevalence of anxiety in teachers, whatever class level they taught, ranged from 10% to 49.4%, and depression from 15.9% to 28.9%. Indeed, the COVID-19 pandemic has intensified the public´s collective emotions as a result of major changes in the society. This includes loneliness and isolation, the loss of loved ones, fear of being contaminated, financial struggles, and concerns about the management of the pandemic [17]. In addition, teachers, who already have stressful and demanding jobs, have to adapt to many additional changes during the pandemic, which could potentially make them more vulnerable to psychological distress [18]. Furthermore, according to Fiorillo and Gorwood [19], people who are significantly exposed to the virus are at risk of mental health and psychosocial repercussions. Teachers' physical safety is seriously threatened by their work environment. Being aware of the serious complications associated with the virus can increase their sense of vulnerability. It has been proved that threat assessments cause excessive fear and that they are associated with psychological distress, in particular, anxiety and depression [20].

The findings of our study indicated a significant association between female gender and the emergence of feelings of anxiety and depression. Our results are consistent with those of relevant studies which proved that women were more likely to show symptoms of anxiety and intense emotional discomfort [21] and that they were more prone to experiencing depression than men during the outbreak [6,18,22]. In a study conducted in South Africa during the second wave of the pandemic (May-July 2021) [23], women reported significantly higher levels of anxiety and hopelessness than men. According to Li *et al*. [22], the prevalence of anxiety during COVID-19 was higher for female teachers than for male teachers. In addition, the study of Desouky *et al*. [24] on Egyptian teachers showed that the female gender was an independent predictor for depression and anxiety. Indeed, female teachers find themselves torn between their huge responsibilities in work as well as in their families [25].

According to our results, anxiety and depression were statistically related to the degree of satisfaction with working conditions. More specifically, teachers who were dissatisfied with their working conditions were four times more likely to develop depressive manifestations than teachers whose satisfaction was high. Indeed, the socio-ecological framework of occupational health highlights the relationship between working conditions and the employees´ health [26]. Teachers' mental health can be shaped by their work environments and life stressors such as the COVID-19 pandemic [27]. Studies have shown that perceived negative working conditions as well as a poor work environment predicted the onset of depressive symptoms in teachers [27,28].

Similarly, to the study of Desouky *et al*. [24], the current study showed that anxiety and depression were significantly more prevalent in teachers with higher workloads. However, given the impact of the pandemic situation, the teachers´ work and its quality requirements are increasingly demanding [29]. Thus, we have to think about preventing burnout syndrome which appears due to chronically high levels of stress, sustained work-related exhaustion, and overwhelming negative emotions [30].

In addition, depression was also independently associated in our sample with the irregular wearing of protective masks. Nevertheless, a study of university students in southwestern Ethiopia [31] demonstrated that wearing a face mask was a predictor of mental health disorders. Along with our findings, in a study exploring depression in Hong Kong using the PHQ-9 scale [32], depression was less likely among those who wore face masks correctly. The result found could be based on the explanation of some authors who showed that wearing a mask could create a false sense of security in some people [33], which could protect them against the development of depressive symptoms.

Our results indicated a statistically significant association between the presence of sleep disturbances during the COVID-19 pandemic and the emergence of anxiety and depression manifestations. Teachers who suffered from insomnia were five times more likely to develop anxious symptoms than teachers without sleep disturbances. In fact, sleep is crucial for human life and it is closely related to emotion. Poor sleep is a risk factor for anxiety [34], particularly in times of COVID-19 [31]. Long-term sleep deprivation is also associated with long-term fatigue and body aches, which in turn can lead to a decline in mental or physical functioning and be strong predictors of depression [35].

In our study, the presence of suicidal thoughts was significantly associated with anxiety and depressive disorders. In China, the rate of teachers with suicidal thoughts or self-injury during the pandemic accounted for 2.8% of the total population [18] (versus 10.4% in our study). Furthermore, studies have proved that affective dysregulated temperaments in workers were linked to both non-suicidal self-injury and suicidal behavior [36]. Finally, in our series, anxiety and depression were strongly and independently associated with each other. Few studies have investigated the association between these two entities in teachers in times of COVID-19. In a study of teachers from South Africa [37], anxiety was positively related to depression (p<0.001). This could be explained by the relationship between work-related stressors in the school environment responsible for the development of both anxiety and depression among teachers [38,39].

However, our study revealed that anxiety and depression had no correlation with age. In the same way, some studies showed no statistical difference in depression severity in terms of age [18]. Other studies confirmed that older age groups had an increased rate of various cardiovascular, auto-immune, and mental disorders. They were also more vulnerable to the organic and mental impacts of the new coronavirus with social distancing and isolation causing a bigger threat due to the likelihood of depression and stress [40,41]. In Egyptian teachers [24], anxiety and depression scores were significantly higher among teachers with an age greater than 40 years. Similarly, in Chinese teachers [18], those over 45 years old were more likely to exhibit symptoms of anxiety. This can partly be justified by the higher morbidity and mortality rates of the new coronavirus among the elderly. Hence, it is not surprising that older people in endemic areas appeared to experience a lower health-related quality of life compared to younger people [42].

The educational level and professional seniority did not seem to be related to the emergence of symptoms of anxiety and depression in our sample. Similarly, several studies [18,42] showed that educational level did not have a relation with anxiety and depressive disorders among teachers during the COVID-19 pandemic. Other studies [24] indicate that anxiety and depression scores were significantly higher among teachers with higher qualifications and higher teaching experience. These latters were independent predictors of depression. In the general population, low education level was one of the main determinants of mental health during the COVID-19 pandemic [43].

Our study showed no association between vaccination status and anxio-depressive symptoms. Nevertheless, studies have shown that increased or decreased levels of emotion might affect one´s motivation and willingness to engage in preventive health behaviors [6,21,44]. The widespread anxiety, loss, and psychological fatigue brought on by the pandemic had a huge impact on health behaviors and vaccination intentions [26,31]. However, our result may be explained by the fact that some people have become emotionally detached due to exhaustion and passivity associated with the adverse, uncontrollable nature of this crisis and its prolonged uncertainty [44,45]. Acknowledging anger, fears, and other negative emotions while highlighting the rigorous safety and efficacy standards of the COVID-19 vaccine development process and promoting individuals´ self-efficacy through vaccination, might be a way to increase vaccine confidence [46].

The results of our study have several important practical implications. They must draw the government's attention to the high rates of anxiety and depression among secondary school teachers in the context of the COVID-19 epidemic and the need to screen for these disorders. The health department has already set up a free, anonymous hotline which is open for any professional with psychosocial suffering from the pandemic. This helpline has a mission of listening, support, guidance, and counseling. This service requires the intervention of psychiatrists and psychologists to enable the expression of emotions and encourage the verbalization of situations experienced and, if necessary, referral to specialised consultations [47]. However, more studies such as ours, screening psychological disorders, especially in the population of secondary school teachers, must be conducted in different secondary schools in Tunisia. The overarching goal is to gain insight on how to help government organizations and healthcare professionals protect the psychological well-being of secondary school teachers in the face of such an epidemic with the hope of providing cognitive-behavioral remediation to teachers suffering from anxiety and depression during and after this pandemic.

In addition, on the basis of our findings, it would be necessary to target factors strongly incriminated in anxiety and depression, such as mask-wearing, dissatisfaction with working conditions, and sleep disturbances, in order to reduce the incidence of these disorders in teachers. In this context, Tunisia's Ministry of Public Health has insisted on wearing of protective masks through its plan of preparation and response to the risk of introduction and dissemination of COVID-19 in Tunisia. This plan was based on wearing masks, physical distancing, activation of control and inspection bodies, application of quarantine, and general confinement [48]. However, during periods of face-to-face education, as was the case for our study, the authorities have given little thought to improving the working conditions of teachers and supplying educational institutions by protective masks which could have improved the teachers´ mental health. Our results suggest therefore the implementation of programs for adjusting working conditions through for example a reduction in the number of working hours, group work every other day, and reorganization of schools by increasing the ventilation of rooms and the distancing of students. As for sleep disturbances, as being a determining factor of anxiety in our sample, they need to be managed through the practice of non-pharmacological interventions such as mindfulness meditation, relaxation, and yoga, which have proved effective in improving sleep quality during this endemic period [49-51]. Yoga offers particularly the convenience of online delivery [49].

Our study has several strengths. To the best of our knowledge, this is the first Tunisian study that focuses on the impact of the COVID-19 epidemic on secondary school teachers. Additionally, it enabled us to explore the psychological distress of this vulnerable population in depth during an unprecedented epidemic and to uncover the associated factors to address. However, there are some limitations in terms of the current research. First, during the COVID-19 epidemic, data could only be obtained through online tools, which could explain the small size of our sample. Secondly, the representativeness of the sample could have been reduced given its reduced size and the sampling bias. Therefore, the results cannot be generalized to the population of teachers in Tunisia. A larger sample size could make the results of this study more obvious.

## Conclusion

The findings of our study indicate a specific profile of Tunisian secondary school teachers during the COVID-19 pandemic in our sample, characterized by a high rate of anxiety and depressive disorders which were related to personal factors such as female gender, sleep disturbances, and suicidal thoughts as well as to professional and epidemic factors which revolve around workload, working conditions, and mask-wearing. Acting on these factors as well as screening and early management of psychiatric disorders may improve the mental health status of secondary school teachers. Nowadays, more studies are needed to assess the mental health of these teachers after the restoration of normal teaching conditions.

### What is known about this topic

- *The increasing number of cases caused by COVID-19 created a climate of alarm and fear due to the very real threat of contraction and potential manifestation of the disease;*
- *This epidemic is the greatest challenge that the expanded national education systems have ever faced;*
- *Teachers, apart from having a stressful and challenging profession, have to adapt during this pandemic time to many other changes that could potentially make them more vulnerable to psychological distress*.

### What this study adds

- *Our sample of Tunisian secondary school teachers during the COVID-19 pandemic was characterized by a high rate of anxiety and depressive disorders;*
- *Anxiety was strongly associated with sleep disturbances and depression while depression was strongly associated with dissatisfaction with working conditions, irregular wearing of protective masks, and the presence of anxiety*.
- *Acting on factors associated with anxiety and depression as well as screening and early management of psychiatric disorders may improve the mental health status of secondary school teachers*.
